# Synthesis of 4-(dimethylamino)pyridine propylthioacetate coated gold nanoparticles and their antibacterial and photophysical activity

**DOI:** 10.1186/s12951-017-0332-z

**Published:** 2018-01-29

**Authors:** Ayaz Anwar, Sadia Khalid, Samina Perveen, Shakil Ahmed, Ruqaiyyah Siddiqui, Naveed Ahmed Khan, Muhammad Raza Shah

**Affiliations:** 1 International Center for Chemical and Biological Sciences, H.E.J. Research Institute of Chemistry, University of Karachi, Karachi, 75270 Pakistan; 2 Department of Biological Sciences, School of Science and Technology, Sunway University, 47500 Subang Jaya, Selangor Malaysia

**Keywords:** Gold nanoparticles, DMAP, Antibacterial activity, *E. coli*, Synergistic effect, AFM

## Abstract

**Background:**

Gold nanoparticles are useful candidate for drug delivery applications and are associated with enhancement in the bioavailability of coated drugs and/or therapeutic agent. Since, heterocyclic compounds are known to exhibit antimicrobial potential against variety of pathogens, we designed this study to evaluate the antibacterial effects of gold nanoparticles conjugation with new synthesized cationic ligand; 4-Dimethyl aminopyridinium propylthioacetate (DMAP-PTA) in comparison with pure compound and antibiotic drug Pefloxacin. Antibacterial activity of DMAP-PTA coated gold nanoparticles was investigated against a fecal strain of *E. coli* (ATCC 8739).

**Results:**

A new dimethyl aminopyridine based stabilizing agent named as DMAP-PTA was synthesized and used for stabilization of gold nanoparticles. Gold nanoparticles coated with DMAP-PTA abbreviated as DMAP-PTA-AuNPs were thoroughly characterized by UV–visible, FT-IR spectroscopic methods and transmission electron microscope before biological assay. DMAP-PTA, DMAP-PTA-AuNPs and Pefloxacin were examined for their antibacterial potential against *E. coli*, and the minimum inhibitory concentration (MIC) was determined to be 300, 200 and 50 µg/mL respectively. Gold nanoparticles conjugation was found to significantly enhance the antibacterial activity of DMAP-PTA as compared to pure compound. Moreover, effects of DMAP-PTA-AuNPs on the antibacterial potential of Pefloxacin was also evaluated by combination therapy of 1:1 mixture of DMAP-PTA-AuNPs and Pefloxacin against *E. coli* in a wide range of concentrations from 5 to 300 µg/mL. The MIC of Pefloxacin + DMAP-PTA-AuNPs mixture was found to be 25 µg/mL as compared to Pefloxacin alone (50 µg/mL), which clearly indicates that DMAP-PTA-AuNPs increased the potency of Pefloxacin. AFM analysis was also carried out to show morphological changes occur in bacteria before and after treatment of test samples. Furthermore, DMAP-PTA-AuNPs showed high selectivity towards Pefloxacin in spectrophotometric drug recognition studies which offers tremendous potential for analytical applications.

**Conclusions:**

Gold nanoparticles conjugation was shown to enhance the antibacterial efficacy of DMAP-PTA ligand, while DMAP-PTA-AuNPs also induced synergistic effects on the potency of Pefloxacin against *E. coli*. DMAP-PTA-AuNPs were also developed as Pefloxacin probes in recognizing the drug in blood and water samples in the presence of other drugs.

**Electronic supplementary material:**

The online version of this article (10.1186/s12951-017-0332-z) contains supplementary material, which is available to authorized users.

## Background

Infectious diseases are one of the leading causes of morbidity and mortality worldwide [1]. Pathogenic *Escherichia coli* (*E. coli*) are gram-negative bacteria which are causative agents of urinary tract infections, neonatal meningitis, hospital acquired septicemia and enteritis in humans [2]. *E. coli* has developed unmatched resistance to drugs as compared to other microbes [3]. Since bacterial resistance is multifactorial, interdisciplinary approaches and new strategies to design more efficient antibacterial agents are urgently required. Recently immense efforts are made to improve the availability of drugs by synergizing them with bio-enhancing agents [4, 5].

Nanotechnology is providing promising platform to develop antibacterial agents such as silica, zinc oxide, silver and gold against gram-negative bacteria (*E. coli*) but their low selectivity and toxicity is still a challenge [6–9]. One way to cope with multi drug resistance bacteria is to co-formulate antibiotics with nanoparticles. Brown et al. [10] showed an effective synergism of ampicillin with noble metal nanoparticles against multi drug resistant strains such as *Pseudomonas aeruginosa*, *Enterobacter aerogenes* and *Staphylococcus aureus* [10]. Various bacteria have developed resistance against Pefloxacin, so enhancing the potency of Pefloxacin with nanoparticle is an attractive strategy to be adopted. Pefloxacin, 1-ethyl-6-fluoro-1, 4-dihydro-7-(4-methylpiperazin-1-yl)-4-oxoquinolone-3-carboxylic acid, is a fluoroquinolone antibacterial agent. Fluoroquinolones are effective against most gram-negative and gram-positive bacteria [11, 12], including some protozoan *Plasmodium falciparum* [13]. It is used for the treatment for skin diseases and various kinds of urinary tract infections [14]. Pefloxacin is also used in aquaculture industry to treat and prevent a variety of infectious diseases, but its overuse runs the risk of increasing drug residues in seafood and contributing to bacterial resistance [15]. In order to enhance anti-bacterial potential of Pefloxacin through synergistic effect of nanoparticles, pyridinium derivative was selected for the coating of AuNPs, since they are abundantly available in natural products and drugs [16]. Their ionic liquids are also used to develop gold nanoparticles for catalysis [17]. Nanoparticles coated with dimethyl amino pyridine [18] are used in drug delivery [19], film formation [20], detection of dopamine in bio samples [21], and are also investigated for their interaction with calf thymus DNA and living cells [22].

DMAP itself has already been used to coat AuNPs, but they have limited stability and antibacterial applications. DMAP-PTA on the other hand provides a flexible thiolated head group for stabilization of nanoparticles, which ensures greater stability to AuNPs and striking chemosensing and antibacterial properties due to different functionality at the surface of nanoparticles. Herein, we report the synthesis of DMAP-PTA for the stabilization of AuNPs to form DMAP-PTA-AuNPs and exploited their ability to enhance the antibacterial potential of Pefloxacin. Furthermore, this protocol provides an easily to reproduce, rapid, economical and sensitive method for selectively recognizing Pefloxacin in tap water.

## Methods

### Chemicals and instruments

All the chemicals used in this study were of analytical grade and used without any pretreatment. Dimethyl aminopyridine was purchased from Tokyo chemicals industry (TCI, China). Dibromo propane and potassium thioacetate were purchased from Alfa-Aesar (MA, USA). Chloroauric acid trihydrate and Pefloxacin were purchased from Sigma-Aldrich (St. Louis, USA). Oven dried glass wares were washed with aqua regia and rinsed by deionized water before using. Freshly prepared solutions were used throughout synthesis of nanoparticles and photophysical analysis. Instruments used for the analysis are UV–visible spectrophotometer (Evolution 300), IR spectrophotometer (Vector 22, Bruker), matrix assisted laser desorption ionization (MALDI) mass spectrometer (Bruker Ultra flex, TOF–TOF), NMR spectrometer (^1^H-NMR 300 MHz and ^13^C NMR 125 MHz) (Bruker, Switzerland), atomic force microscope (AFM) (Agilent 5500), and transmission electron microscope (Hi-Tech, 300 kV TEM H-9500).

### Synthesis of ligand DMAP-PTA

**Scheme 1:**
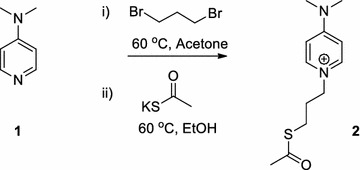
Synthesis of ligand DMAP-PTA

DMAP (**1**) (245 mg, 2 mol) and Dibromopropane (1.6 mL, 16 mol) were stirred in 5 mL acetone at 60 °C for 30 min. Solvent was evaporated under vacuum to obtain white precipitates of the mono substituted product 1-(3-bromopropyl)-4-(dimethylamino)pyridin-1-ium. The precipitates (243 mg, 1 mol) were dissolved in 5 mL ethanol and potassium thioacetate (140 mg, 1.2 mol) was added. The reaction mixture was stirred at 60 °C, while reaction progress was monitored by thin layer chromatography (TLC; dichloromethane: methanol 9:1). After 10 h the solvent was evaporated under reduced pressure to obtain brown solid crystalline residue which were filtered and washed with 10 mL of methanol (Scheme 1). Methanol filtrate was evaporated to get the N-alkylated product DMAP-PTA in white crystalline state and was characterized by mass and NMR spectroscopy. Yield 442 mg, 92%. ESI–MS 277.07 KM^+^. Melting point 240–242 °C. ^1^H-NMR in MeOD, 8.16 (d, 2H, Ar, meta), 6.99 (d, 2H, Ar, ortho), 4.21 (t, 2H, N–CH_2_), 3.29 (s, 6H, N–CH_3_), 2.86 (t, 2H, S–CH_2_), 2.10 (m, 2H, C–CH_2_–C), 1.89 (s, 3H, CH_3_CO). ^13^C-NMR in MeOD, 180.30 (Ar, ipso), 143.21 (Ar, meta), 109.01 (Ar, ortho), 57.21 (CH_2_–N), 40.34 (CH_2_–S), 32.13 (CH_3_–CO), 31.46 (CH_3_–N), 24.14 (CH_2_–CH_2_–CH_2_).

### Synthesis and stabilization of DMAP-PTA-AuNPs

1 mL of 0.1 mM solution of DMAP-PTA was added in a shaking solution of 10 mL of chloroauric acid trihydrate (0.1 mM). After 15 min 0.1 mL of freshly prepared 4 mM NaBH_4_ solution was added in the reaction mixture. The colorless reaction mixture was turned into clear wine red solution instantly after mixing with NaBH_4_ solution and the reaction mixture was kept on shaking for 2 more hours. The resulting solution was subjected to UV–visible spectrophotometry and TEM imaging.

### Minimum inhibitory concentration

To calculate minimum inhibitory concentration (MIC), the agar well diffusion method was employed. Estimation of minimum inhibitory concentration of Pefloxacine, DMAP-PTA, DMAP-PTA-AuNPs and Pefloxacin mixed with DMAP-PTA-AuNPs was measured against *E. coli* (ATCC 8739). In brief, nutrient agar was used as medium to grow a lawn of *E. coli* at the concentration of 10^6^ cells. Duplicate dilutions were used to calculate minimum inhibition zones. The 60 mm well was made by using a borer. The 1000 µg/mL stock solution of drug, nanoparticles and DMAP-PTA were used to avoid nonspecific merged zones of inhibition. In each well different amounts of various concentrations ranging from 500 to 5 µg/mL were added. The plates were incubated at room temperature for 2 h to allow the diffusion process to take place before it was incubated for 24–48 h at 37 °C ± 1. The zones of inhibition were measured by using a millimeter scale.

### Atomic force microscopy imaging

*Escherichia coli* ATCC 8739 was developed on tryptic soy agar (Oxoid UK) at 37 °C for 24 h in static conditions, which were marked as stock *E. coli* culture. A freshly incubated culture of *E. coli* on tryptic soy agar (Oxoid UK) was diluted in distilled water to make 10^6^ cfu of *E. coli*, and 10 µL droplets of this solution were transferred onto a freshly cleaved mica surface and left to dry. The samples were rinsed several times with Milli-Q water after deposition and air-dried at 25 °C. The dried samples were characterized by atomic force microscopy for morphology and size. The test samples were added into vials of distilled water containing 10^6^ cfu of *E. coli* and incubated for 2 h at 37 °C. All experiments were performed with duplicate cultures for each sample. The same procedure was applied to monitor the melting of *E. coli* after incubations with test samples. All samples were treated with 10^6^ cfu of *E. coli* using above protocol and were analyzed by atomic force microscopy to monitor the effectiveness of these samples. High frequency Si cantilever of 125 µm length, force constant 42 Nm^−1^ and resonance frequency 330 kHz were used. All the samples were prepared and analyzed under same conditions.

## Results

### Synthesis of DMAP-PTA-AuNPs

**Fig. 1:**
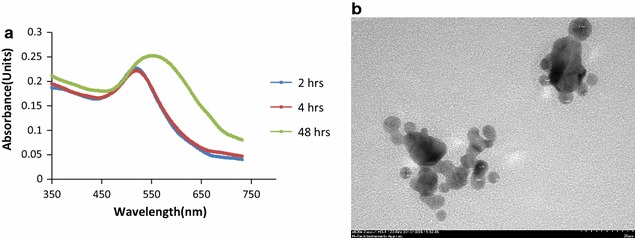
**a** UV–visible spectrum of the DMAP-PTA-AuNPs at different time intervals showing surface plasmon resonance band at 520 nm. DMAP-PTA-AuNPs tend to aggregate after 48 h as can be observed by surface plasmon resonance band’s broadening. **b** TEM image of the DMAP-PTA-AuNPs showing polydispersed particles in the range of 5–20 nm

The DMAP-PTA-AuNPs were synthesized by reduction of chloroauric acid trihydrate with NaBH_4_. Optimized conditions for the synthesis of DMAP-PTA-AuNPs were achieved after varying the ratio of gold salt and DMAP-PTA along with stirring time. Prominent surface plasmon resonance (SPR) band was obtained at 520 nm in UV–visible spectrum by mixing gold salt and DMAP-PTA at 10:1 ratio respectively for 2 h under constant stirring. Larger reaction time (48 h) as shown in Fig. 1a caused aggregation of the DMAP-PTA-AuNPs. The TEM analysis revealed spherical, polydispersed DMAP-PTA-AuNPs with size in the range of 5–20 nm (Fig. 1b).

### Stability of DMAP-PTA-AuNPs

Thermal stability of DMAP-PTA-AuNPs was evaluated by boiling it at 100 °C for 1 h. Slight increase in intensity of SPR band was evident however dispersity of the colloids was unchanged even on heating for longer time (Additional file 1: Figure S1a). This effect in the SPR band maxima is explained by electron dephasing mechanism in the literature [23]. Furthermore, chloride promoted aggregation of the nanoparticles was also studied. Additional file 1: Figure S1b shows that the DMAP-PTA-AuNPs are stable over wide range of concentration of sodium chloride (0.01–10 mM) with the decrease in intensity of SPR band. SPR band broadening and aggregation of the DMAP-PTA-AuNPs was observed after mixing with 50 mM concentration of NaCl.

### Antibacterial analysis of DMAP-PTA-AuNPs against *E. coli* and synergistic effect with Pefloxacin

**Fig. 2:**
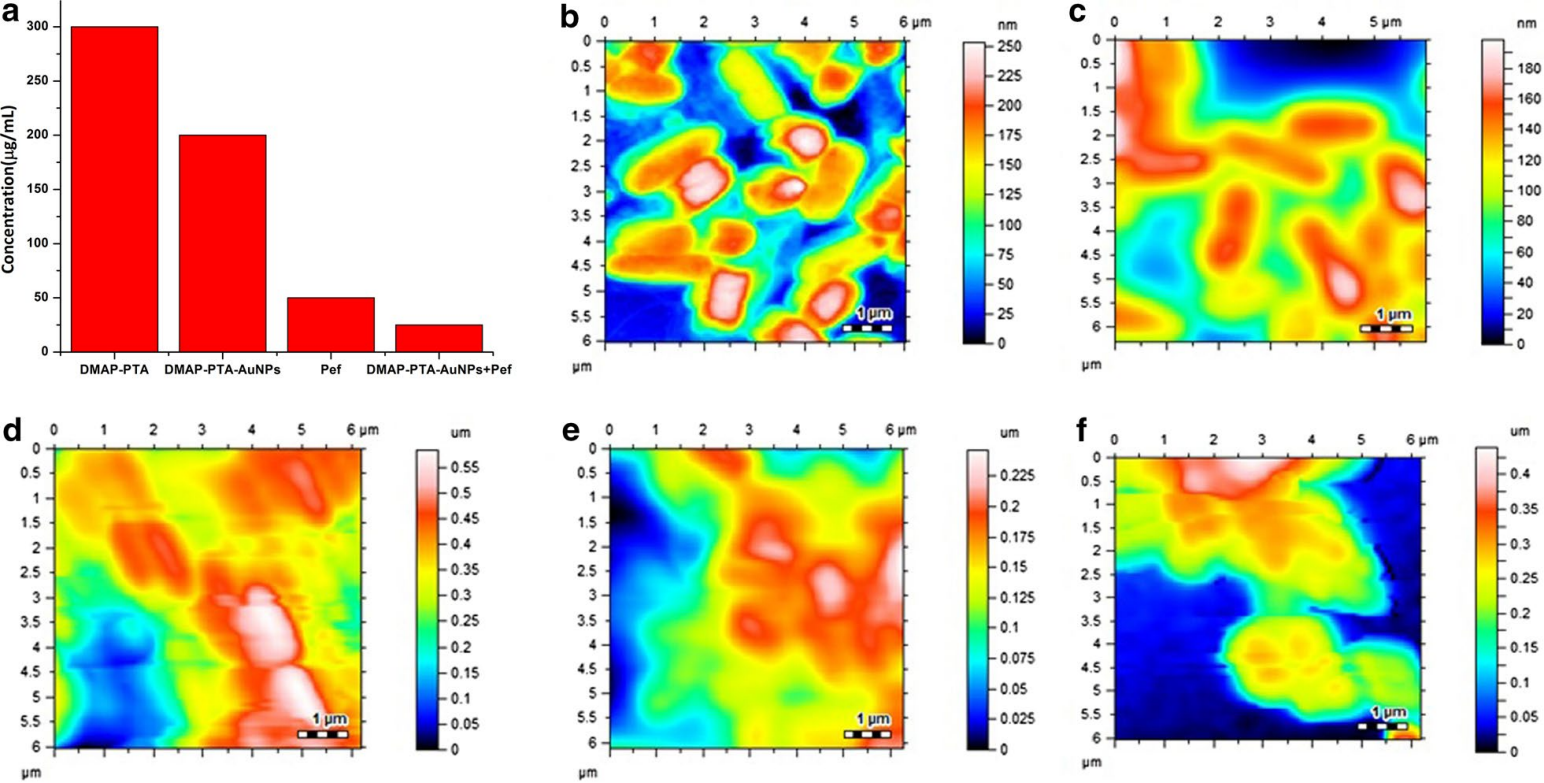
Antibacterial activity of test samples against *E. coli*. **a** Representation of minimum inhibitory concentration; AFM images of *E. coli* (**b**) control (**c**) incubated with DMAP-PTA (**d**) incubated with DMAP-PTA-AuNPs (**e**) incubated with Pefloxacin (**f**) incubated with supramolecular complex of DMAP-PTA-AuNPs with Pefloxacin

The antibacterial activity of DMAP-PTA, Pefloxacin and synergistic effect of DMAP-PTA-AuNPs on Pefloxacin was investigated at various doses. In each well different concentration of DMAP-PTA-AuNPs, Pefloxacin, DMAP-PTA and mixture of DMAP-PTA-AuNPs with Pefloxacin ranging from 5 to 300 µg/mL were added and the minimum inhibitory concentrations were determined. The results of MICs are summarized in Fig. 2a.

Along with the conventional agar well diffusion assay, AFM based assay was also used to evaluate the antibacterial effect of tested samples at MICs on the morphology of *E. coli*. AFM analysis as shown in Fig. 2b–f also supported the results obtained from the zone of inhibition assay.

### Selective recognition of Pefloxacin by DMAP-PTA-AuNPs

**Fig. 3:**
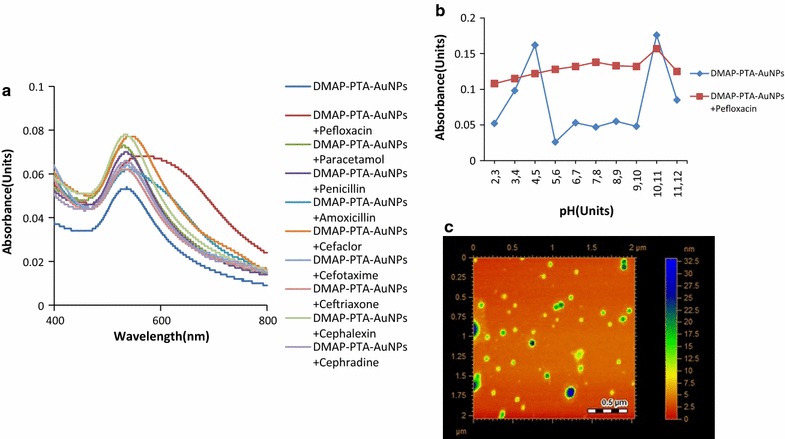
**a** UV–visible screening of tested drugs by DMAP-PTA-AuNPs. **b** Effect of pH on the interactions of DMAP-PTA-AuNPs with pefloxacin. **c** AFM image scanned for the supramolecular complex of DMAP-PTA-AuNPs with Pefloxacin (1:1)

The affinity of DMAP-PTA-AuNPs for various drugs was assessed through UV–visible spectroscopy by mixing 1:1 v/v mixture of 0.1 mM various drugs solutions and DMAP-PTA-AuNPs. The interactions of DMAP-PTA-AuNPs with tested drugs such as Pefloxacin, Paracetamol, Penicillin, Amoxicillin, Cefaclor, Cefotaxime, Ceftriaxone, Cephalexin, and Cephradine was investigated (Fig. 3a). The interactions of drugs with DMAP-PTA-AuNPs lead to enhancement of its absorbance intensity when examined through UV–visible spectrometer except Pefloxacin where red shift in the SPR band of DMAP-PTA-AuNPs was observed providing absorption maxima at 700 nm.

Since the pH of the medium has a great impact on the supramolecular interactions of host and guest so an optimum pH was figured out for the interactions of DMAP-PTA-AuNPs with Pefloxacin. As shown in Fig. 3b that maximum interactions between DMAP-PTA-AuNPs and Pefloxacin was observed at pH 10–11, however considerable interactions of DMAP-PTA-AuNPs with Pefloxacin can be seen in other pHs as well. As mentioned earlier that the mixing of Pefloxacin with DMAP-PTA-AuNPs induced aggregations in DMAP-PTA-AuNPs which is also supported by AFM analysis. As shown in Fig. 3c that large aggregates (50–150 nm) of DMAP-PTA-AuNPs formed after mixing with Pefloxacin.

**Fig. 4:**
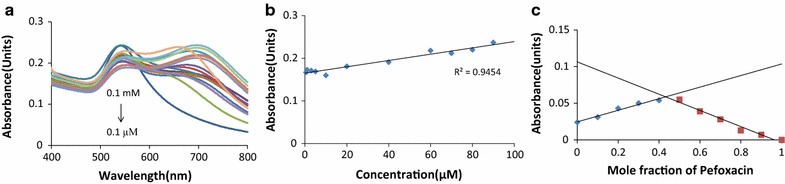
**a** Titration of Pefloxacin against DMAP-PTA-AuNPs showing increase in the absorption intensity of band appeared at 700 nm by increment in concentration of Pefloxacin. **b** Calibration curve at 700 nm, **c** Job’s plot analysis to establish binding stoichiometry between DMAP-PTA-AuNPs and Pefloxacin

The optical response of DMAP-PTA-AuNPs towards Pefloxacin was recorded by titrating Pefloxacin against fixed concentration of DMAP-PTA-AuNPs and changes in the intensity SPR band at 700 nm was monitored (Fig. 4a). As shown in Fig. 4b that the titration follows beer’s law and exhibited a good linear correlation in the range from 0.1 to 90 μM with the regression constant (R^2^) equal to 0.945 (Fig. 4b). The limit of detection (LOD) and limit of quantification (LOQ) were found to be 1.5 and 4.8 μM respectively. The fluctuation in titration curves of SPR band suggests the formation of different extent of aggregates with altering Pefloxacin concentration. Therefore to find out the exact binding stoichiometry between supramolecular complexes of DMAP-PTA-AuNPs with Pefloxacin, Job’s plot method was utilized. Absorption spectra were recorded by varying the mole ratio of Pefloxacin with respect to DMAP-PTA-AuNPs. The minimum on the plot between absorbance and mole fraction appeared at 0.5, which correspond to the 1:1 stoichiometric relation between the stable DMAP-PTA-AuNPs-Pefloxacin complex (Fig. 4c).

### Evaluation of specific recognition of Pefloxacin by DMAP-PTA-AuNPs

**Fig. 5:**
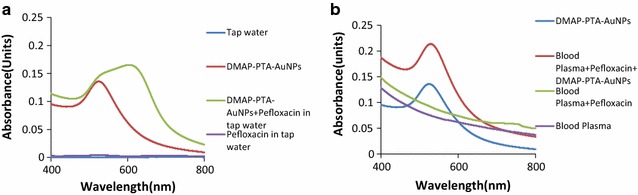
Selective recognition of Pefloxacin by DMAP-PTA-AuNPs in **a** tap water, **b** Human blood plasma

The interfering effect from the biological matrix like blood plasma and ingredients of water on the supramolecular association of DMAP-PTA-AuNPs with Pefloxacin was also evaluated. Human blood plasma and tap water samples were spiked with 0.1 mM concentration of Pefloxacin and then titrated against DMAP-PTA-AuNPs. Figure 5a shows that the recognition of Pefloxacin works precisely well in tap water real sample, however the complex ingredients of blood plasma seem to interfere with the interactions of DMAP-PTA-AuNPs with Pefloxacin thus the red shift is not observed (Fig. 5b).

## Discussion

The present study was aimed to develop the newly synthesized DMAP-PTA-AuNPs as an antibacterial agent. Ligand DMAP-PTA was screened against *E. coli* ATCC 8739 using classical agar well diffusion antibacterial bioassay and atomic force microscopic guided assay using Pefloxacin as standard. The selective recognition and synergistic effect of DMAP-PTA-AuNPs on Pefloxacin was investigated through classical biological assay, AFM based assay and spectral analysis. Since antibiotics are the first line of defense against most of the microbial infections hence bacterial resistance to antibiotics is a problem of prime concern that limits therapeutic options and demands newer approaches. Nanoparticles are promising alternate for the enhanced and targeted provision of drugs against certain microbes [24]. The newly synthesized compound DMAP-PTA is potent and can stabilize gold nanoparticles (DMAP-PTA-AuNPs), that selectivity recognizes Pefloxacin with long term stability, rapid spectrophotometric response and tolerance over a broad range of pH (2–12).

Supramolecular interactions of DMAP-PTA-AuNPs with Pefloxacin were also supported by antibacterial assay and this interaction is very much prone to interference from biological matrix of bacterial cultures as well as a number of ingredients in water and blood plasma. In order to eliminate the false positive results induced by these ingredients on the antibacterial potential of Pefloxacin, we carried out systematic molecular recognition studies to evaluate the interactions of Pefloxacin with DMAP-PTA-AuNPs and factors affecting these interactions.

The successful formation of DMAP-PTA-AuNPs was also verified by FT-IR spectroscopy analysis (Additional file 1: Figure S2). Careful comparatively analysis of the FT-IR spectrum of DMAP-PTA and DMAP-PTA-AuNPs suggested the cleavage of thioester functionality and formation of thiolate which stabilized the gold nanoparticles as the thioester peak at 1716 cm^−1^ in the FT-IR spectrum of DMAP-PTA disappeared after conjugation with nanoparticles while the rest of the characteristics peak for DMAP remained unchanged. The interaction of DMAP-PTA-AuNPs with Pefloxacin was also investigated through FT-IR spectroscopy. The FT-IR spectrum of Pefloxacin revealed stretching for O–H at 3427, N–CH_3_ at 2646, carboxyl carbonyl at 1716, C–O at 1627, C–F at 1091 cm^−1^ while asymmetric and symmetric stretches for sulfoxide were observed at 1197 and 1051 cm^−1^ [25]. The mixing of Pefloxacin with DMAP-PTA-AuNPs accompanied disappearance of carbonyl peak at 1716 cm^−1^ of Pefloxacin appearance of a new peak at 1583 cm^−1^ attributed to the carboxylate moiety, which is believed to be responsible for the aggregation of DMAP-PTA-AuNPs via hydrogen bonding.

It is clearly evident from Fig. 2a that compound DMAP-PTA alone remained inactive up to concentration of 300 µg/mL against *E. coli*, while the MIC of DMAP-PTA-AuNPs and Pefloxacin was found to be 200 and 50 µg/mL respectively. The bare AuNPs were found to be inactive in our earlier reports and here as well against *E. coli* [8]. Interestingly 1:1 mixture of DMAP-PTA-AuNPs and Pefloxacin revealed MIC of 25 µg/mL against *E. coli* suggesting the synergizing role of DMAP-PTA-AuNPs on the antibacterial potential of Pefloxacin. Similarly, Fig. 2a also concludes that conjugation of DMAP-PTA to AuNPs resulted in enhancement of the antibacterial activity of ligand.

AFM images of untreated *E. coli* (control) showed a relatively smooth surface of the bacteria without indentations (Fig. 2b). Slight changes in the *E. coli* morphology was observed as compared to the untreated cells (Fig. 2c). The AFM images showed that DMAP-PTA-AuNPs (200 µg/mL) inhibit the growth of *E. coli* more effectively than DMAP-PTA (300 µg/mL). The DMAP-PTA-AuNPs induced aggregation and melting (Fig. 2d) of *E. coli* while Pefloxacin (50 µg/mL) also caused degradation of *E. coli* (Fig. 2e). Significant morphological changes in the *E. coli* were observed when 25 µg/mL concentration of supramolecular complex of Pefloxacin with DMAP-PTA-AuNPs (Fig. 2f) were incubated with it. Since Pefloxacin triggered morphological changes in the *E. coli* at concentration of 50 µg/mL while of supramolecular complex of Pefloxacin with DMAP-PTA-AuNPs in 1:1 ratio induced morphological changes in the *E. coli* at concentration of 25 µg/mL under identical conditions while DMAP-PTA-AuNPs and Pefloxacin remained inactive against *E. coli* when incubated separately with concentration of 25 µg/mL. This it can safely be concluded that DMAP-PTA-AuNPs is synergizing the anti-bacterial potential of Pefloxacin.

Figure 3b shows that the nascent pH of DMAP-PTA-AuNPs which was found to be in the range of 4–5 due cationic nature of ligand coated around nanoparticle. The DMAP-PTA-AuNPs was found to be stable in both basic media, as well as acidic media, interesting aggregations of the DMAP-PTA-AuNPs was observed at neutral pH as indicated by broadening of the SPR band. It can safely be concluded that Pefloxacin interacts with DMAP-PTA-AuNPs in broad range of pHs.

As mentioned earlier that DMAP-PTA-AuNPs induced synergistic effect on the anti-bacterial activities of Pefloxacin through supramolecular interactions. Since a number of other ingredients in the bacterial culture, water and blood plasma can also induce synergism on the anti-bacterial properties of Pefloxacin so it is essential to establish that the ingredients from blood plasma, bacterial culture and water are not interfering in the supramolecular interaction of DMAP-PTA-AuNPs and Pefloxacin. Therefore selective discrimination of Pefloxacin by DMAP-PTA-AuNPs over other drugs was investigated. Competing drugs such as 6-amino penicillinic acid, amoxicillin, aspirin, cefaclor, diclofenac sodium, flurbiprofen, paracetamol, and penicillin were mixed with 1:1 (v/v) mixture of Pefloxacin and DMAP-PTA-AuNPs in equivalent ratio. The interference effect of other drugs on the interactions of DMAP-PTA-AuNPs with Pefloxacin was monitored with UV–visible spectroscopy as shown in Additional file 1: Figure S3. The SPR band of DMAP-PTA-AuNPs at 700 nm formed after interaction with Pefloxacin remained unaltered after mixing of the aforementioned drugs in equivalent ratio with DMAP-PTA-AuNPs and Pefloxacin supramolecular complex (with some fluctuation in the case of amoxicillin only), which implies that other drugs did not interfere in the synergistic effect of DMAP-PTA-AuNPs on the anti-bacterial activities.

## Conclusions

In conclusion, dimethyl aminopyridine thiolated compound DMAP-PTA was synthesized and coated on gold nanoparticles (DMAP-PTA-AuNPs). Nanoparticles conjugation increased antibacterial activity of DMAP-PTA. Pefloxacin in combination with DMAP-PTA-AuNPs showed synergistic effects independent of matrix interference. The synergistic effect of DMAP-PTA-AuNPs led to reduction in the MIC of Pefloxacin by at least one order of magnitude. Pefloxacin alone was found to be ineffective at 25 µg/mL, but revealed bactericidal effects against *E. coli* when combined with DMAP-PTA-AuNPs. Furthermore, Pefloxacin showed selective interaction with DMAP-PTA-AuNPs resulting into aggregation of DMAP-PTA-AuNPs as revealed by AFM and UV–visible spectrophotometry. DMAP-PTA-AuNPs exhibited excellent selectivity and specificity towards Pefloxacin over other interfering species, while the pH tolerance allows using this supramolecular complex in almost any environment. The optical response of Pefloxacin towards DMAP-PTA-AuNPs is distinctive and follows linear correlation in a wide range of concentration. Altogether, co-formulations of antibiotics with nanoparticles provide one excellent option to counter the unresolved problem of an increasing resistance of pathogenic bacteria against common antibiotics. Similarly, the combination of antibiotic with nanoparticles could be a realistic approach to decrease the amount of antibiotics currently used in curing the diseases.

## Additional file

**Additional file 1: Figure S1.** (a) Effect of temperature on the stability of DMAP-PTA-AuNPs (Blue curve: Spectrum recorded at 25 ^°^C, Red curve: Spectrum recorded at 100 ^°^C) (b) Salt aggregation study of DMAP-PTA-AuNPs. **Figure S2.** FT-IR spectrum of (a) ligand DMAP-PTA (b) DMAP-PTA-AuNPs (c) DMAP-PTA-AuNPs + Pefloxacin complex. **Figure S3.** Effect of competing drugs on Pefloxacin response of DMAP-PTA-AuNPs.
