# Supplementary material for: Synthesis of 4-(dimethylamino)pyridine propylthioacetate coated gold nanoparticles and their antibacterial and photophysical activity
Source: J Nanobiotechnology. 2018 Jan 29;16:6. doi: 10.1186/s12951-017-0332-z (PMC5787922; doi:10.1186/s12951-017-0332-z)
Supplement: Supplementary file 1 — Additional file 1: Figure S1. (a) Effect of temperature on the stability of DMAP-PTA-AuNPs (Blue curve: Spectrum recorded at 25 °C, Red curve: Spectrum recorded at 100 °C) (b) Salt aggregation study of DMAP-PTA-AuNPs. Figure S2. FT-IR spectrum of (a) ligand DMAP-PTA (b) DMAP-PTA-AuNPs (c) DMAP-PTA-AuNPs + Pefloxacin complex. Figure S3. Effect of competing drugs on Pefloxacin response of DMAP-PTA-AuNPs. [file 12951_2017_332_MOESM1_ESM.doc]

Additional file

**Synthesis of 4-(Dimethylamino)pyridine propylthioacetate coated Gold nanoparticles and their antibacterial and photophysical activity**

Ayaz Anwar^1,2^ *, Sadia Khalid^1^, Samina Perveen^1^, Shakil Ahmed^1^, Ruqaiyyah Siddiqui^2^, Naveed Ahmed Khan^2^ and Muhammad Raza Shah^1^

*1. International Center for Chemical and Biological Sciences, H.E.J. Research Institute of Chemistry, University of Karachi, Karachi 75270, Pakistan*

*2. Department of Biological Sciences, School of Science and Technology, Sunway University, Subang Jaya 47500, Selangor, Malaysia*

*** Address for correspondence:**

**Dr. Ayaz Anwar**

Department of Biological Sciences

School of Science and Technology

Sunway University, Subang Jaya 47500, Selangor, Malaysia.

E-mail: [ayazanwarkk@yahoo.com](mailto:ayazanwarkk@yahoo.com) [ayazanwar@sunway.edu.my](mailto:ayazanwar@sunway.edu.my)

Tel: +603-74918622 Ext. 7189

Fax: +603-56358630

**S1: Stability of DMAP-PTA-AuNPs**


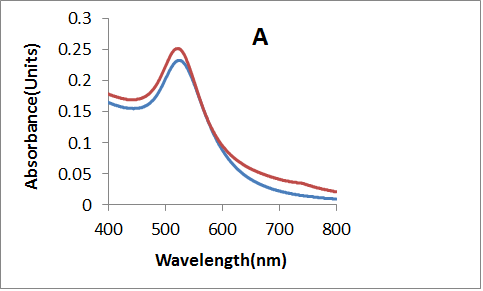


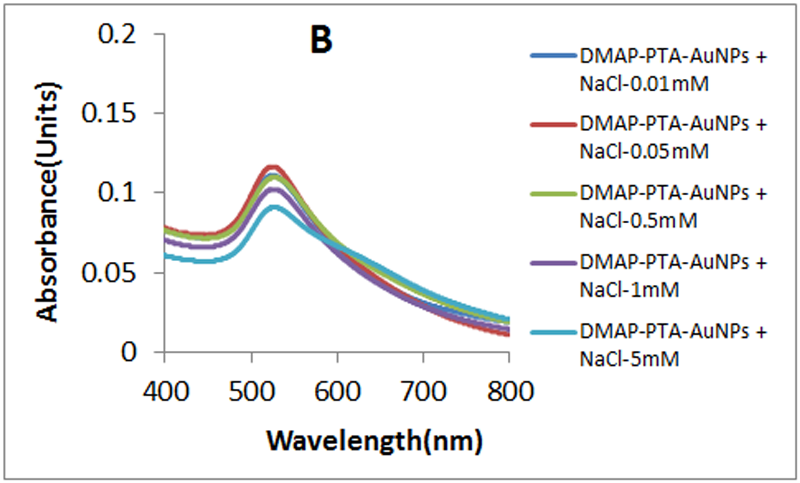


**Figure S1** (a) Effect of temperature on the stability of DMAP-PTA-AuNPs (Blue curve: Spectrum recorded at 25 ^o^C, Red curve: Spectrum recorded at 100 ^o^C) (b) Salt aggregation study of DMAP-PTA-AuNPs

**S2: FT-IR spectral analysis**


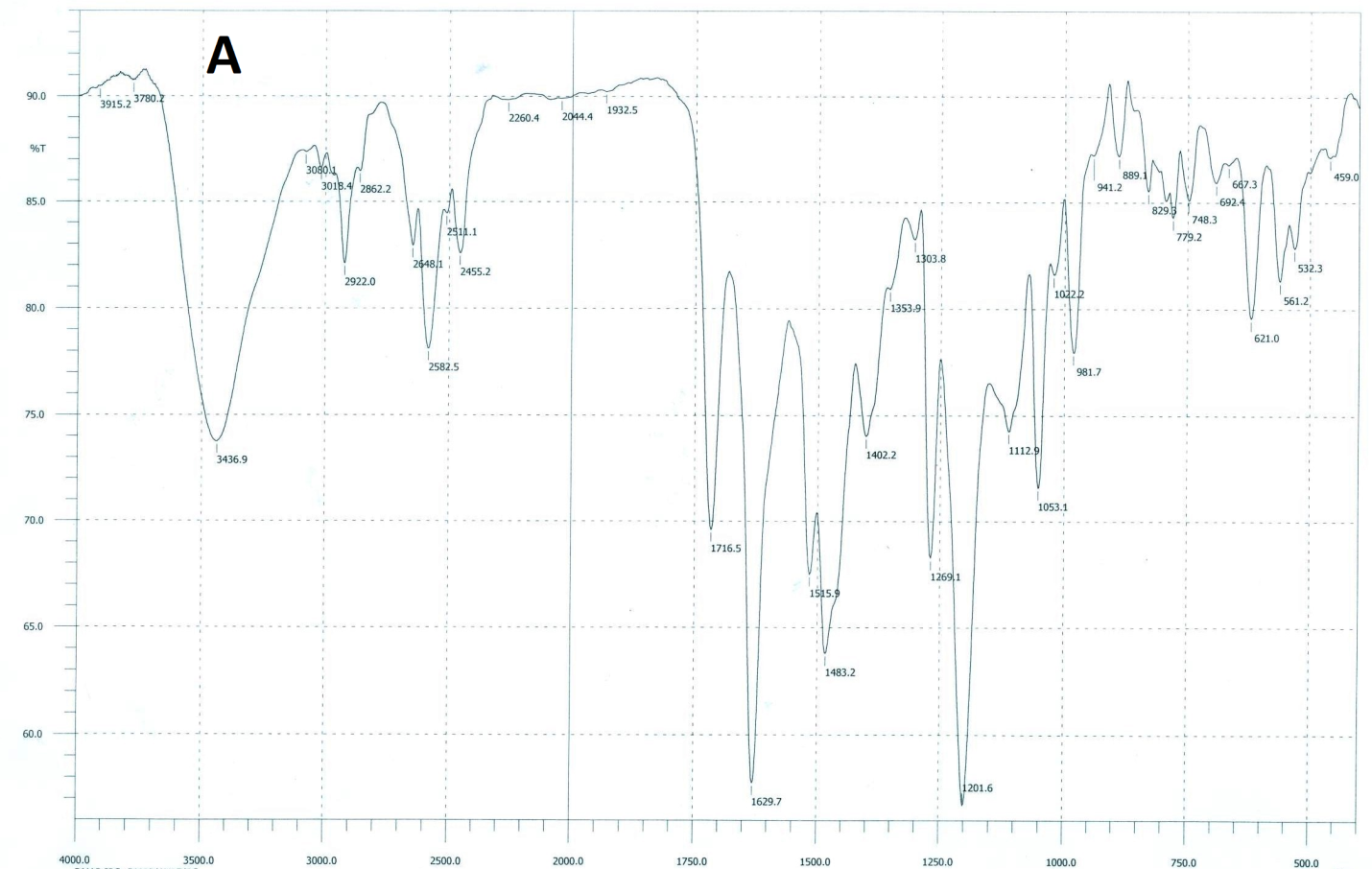


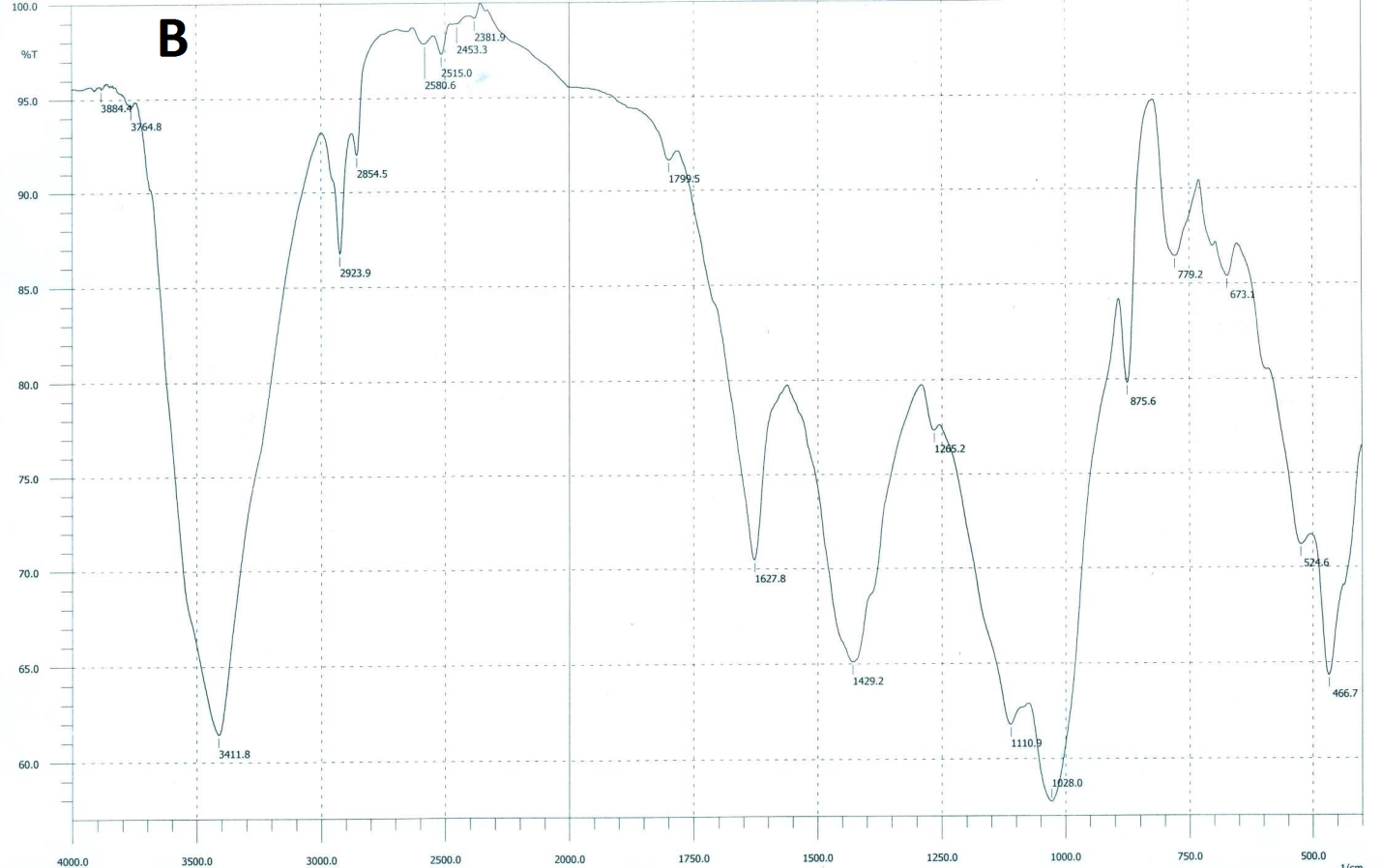


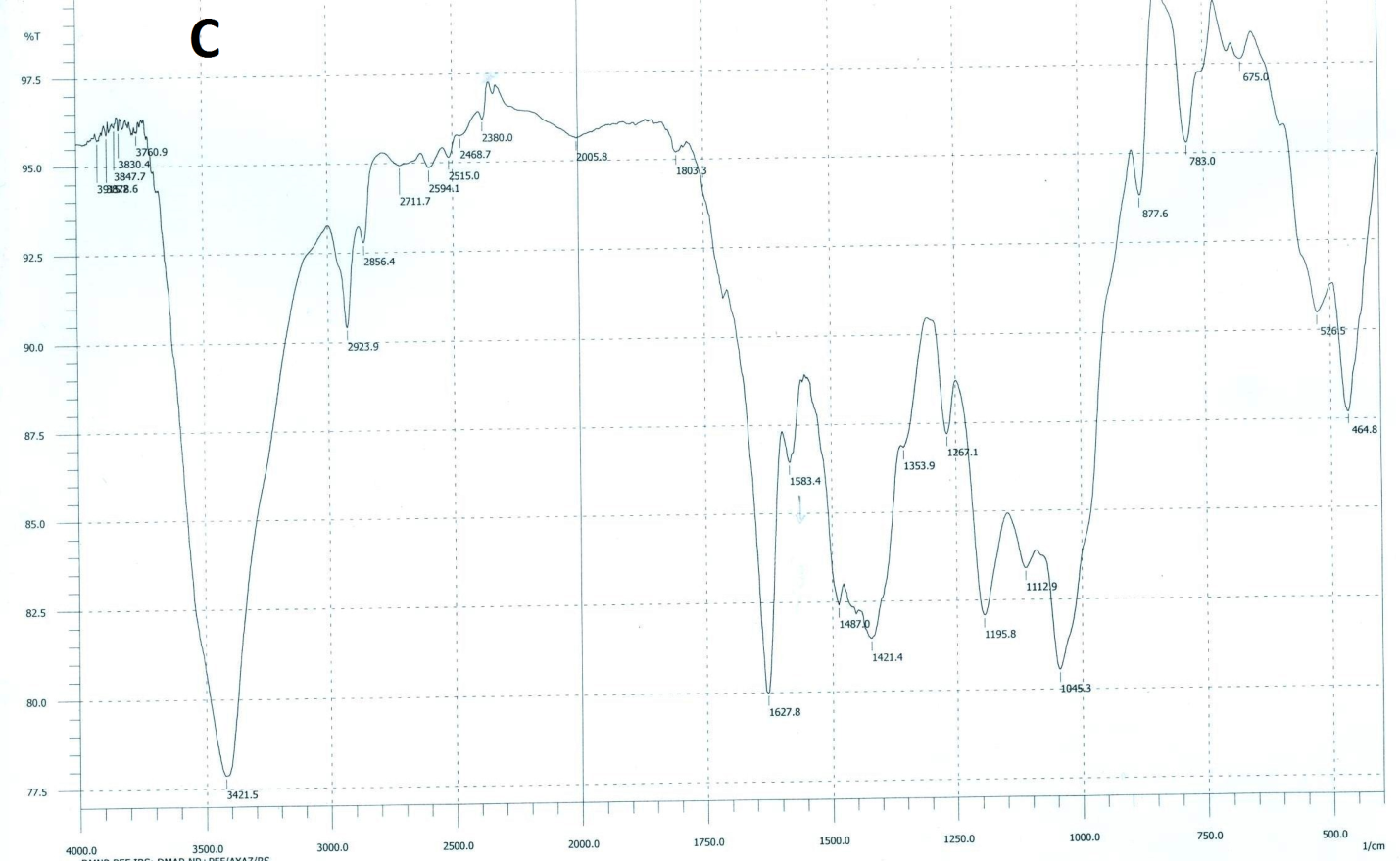


**Figure S2** FT-IR spectrum of (a) ligand DMAP-PTA (b) DMAP-PTA-AuNPs (c) DMAP-PTA-AuNPs+Pefloxacin complex

**S3: Interference study**


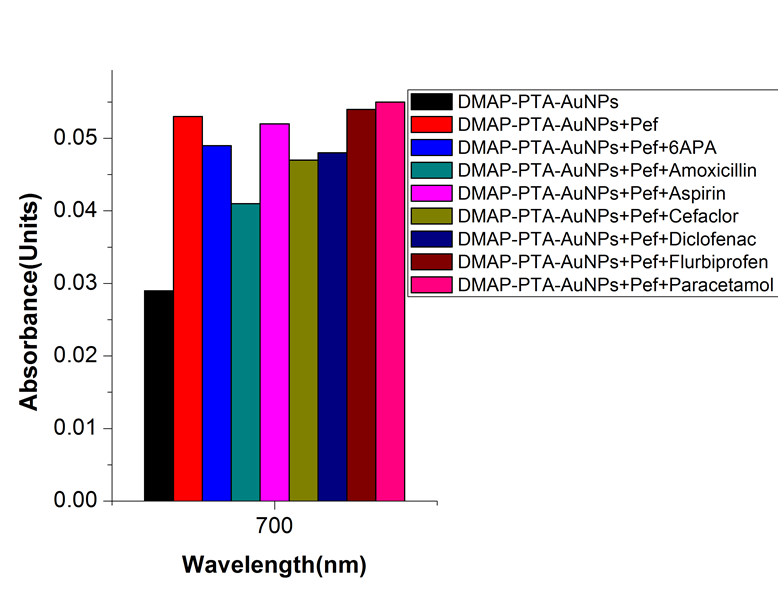


**Figure S3** Effect of competing drugs on Pefloxacin response of DMAP-PTA-AuNPs
